# Osimertinib plus platinum–pemetrexed in newly diagnosed epidermal growth factor receptor mutation-positive advanced/metastatic non-small-cell lung cancer: safety run-in results from the FLAURA2 study

**DOI:** 10.1016/j.esmoop.2021.100271

**Published:** 2021-09-17

**Authors:** D. Planchard, P.-H. Feng, N. Karaseva, S.-W. Kim, T.M. Kim, C.K. Lee, A. Poltoratskiy, N. Yanagitani, R. Marshall, X. Huang, P. Howarth, P.A. Jänne, K. Kobayashi

**Affiliations:** 1 Institut Gustave Roussy, Department of Medical Oncology, Thoracic Oncology Unit, Villejuif, France; 2 Shuang Ho Hospital, Taipei Medical University, Taipei, Taiwan; 3 City Clinical Oncology Dispensary, St. Petersburg, Russia; 4 Asan Medical Center, Seoul, Republic of Korea; 5 Seoul National University Hospital, Seoul, Republic of Korea; 6 Clinical Research Unit, Division of Cancer Services, St. George Hospital, Kogarah, Australia; 7 Department of Clinical Trials, Petrov Research Institute of Oncology, St. Petersburg, Russia; 8 Department of Thoracic Medical Oncology, The Cancer Institute Hospital, Japanese Foundation for Cancer Research, Koto-ku, Tokyo, Japan; 9 AstraZeneca, Cambridge, UK; 10 Dana-Farber Cancer Institute, Boston, USA; 11 Department of Respiratory Medicine, Saitama Medical School International Medical Center, Saitama, Japan

**Keywords:** osimertinib, chemotherapy, lung cancer, EGFRm, safety

## Abstract

**Background:**

The phase III FLAURA2 (NCT04035486) study will evaluate efficacy and safety of first-line osimertinib with platinum–pemetrexed chemotherapy versus osimertinib monotherapy in epidermal growth factor receptor mutation-positive (EGFRm) advanced/metastatic non-small-cell lung cancer (NSCLC). The safety run-in, reported here, assessed the safety and tolerability of osimertinib with chemotherapy prior to the randomized phase III evaluation.

**Patients and methods:**

Patients (≥18 years; Japan: ≥20 years) with EGFRm locally advanced/metastatic NSCLC received oral osimertinib 80 mg once daily (QD), with either intravenous (IV) cisplatin 75 mg/m^2^ or IV carboplatin target area under the curve 5, plus pemetrexed 500 mg/m^2^ every 3 weeks (Q3W) for four cycles. Maintenance was osimertinib 80 mg QD with pemetrexed 500 mg/m^2^ Q3W until progression/discontinuation. The primary objective was to evaluate safety and tolerability of the osimertinib–chemotherapy combination.

**Results:**

Thirty patients (15 per group) received treatment [Asian, 73%; female, 63%; median age (range) 61 (45-84) years]. Adverse events (AEs) were reported by 27 patients (90%): osimertinib–carboplatin–pemetrexed, 100%; osimertinib–cisplatin–pemetrexed, 80%. Most common AEs were constipation (60%) with osimertinib–carboplatin–pemetrexed and nausea (60%) with osimertinib–cisplatin–pemetrexed. In both groups, 20% of patients reported serious AEs. No specific pattern of AEs leading to dose modifications/discontinuations was observed; one patient discontinued all study treatments including osimertinib due to pneumonitis (study-specific discontinuation criterion). Hematologic toxicities were as expected and manageable.

**Conclusions:**

Osimertinib–chemotherapy combination had a manageable safety and tolerability profile in EGFRm advanced/metastatic NSCLC, supporting further assessment in the FLAURA2 randomized phase.

## Highlights

• FLAURA2 aims to assess efficacy and safety of first-line osimertinib with platinum–pemetrexed in EGFRm advanced NSCLC.
• In the FLAURA2 safety run-in period, 30 patients received osimertinib and pemetrexed with carboplatin or cisplatin.
• Most common AEs were constipation and nausea; no AE patterns leading to dose modifications/discontinuations were observed.
• The FLAURA2 safety run-in study showed that the safety profile of this combination was as expected and manageable.

## Introduction

Osimertinib is a third-generation, irreversible, oral epidermal growth factor receptor (EGFR)-tyrosine kinase inhibitor (TKI) that selectively inhibits both EGFR-TKI-sensitizing and EGFR T790M resistance mutations, and has shown efficacy in patients with non-small-cell lung cancer (NSCLC) who have central nervous system (CNS) metastases.^1^, ^2^, ^3^, ^4^ In the phase III FLAURA study, osimertinib demonstrated superiority versus comparator EGFR-TKIs (erlotinib/gefitinib) with respect to progression-free survival and overall survival in patients with previously untreated EGFR mutation-positive [EGFRm; exon 19 deletion (ex19del)/L858R] advanced NSCLC.^4^^,^^5^ This provided the support for osimertinib as the preferred standard of care for first-line treatment of EGFRm NSCLC; however, patients are still likely to acquire resistance.^6^ Further treatment options are required in this setting.

Preclinical data suggest that EGFR-TKIs combined with chemotherapy may act synergistically to restrict the development of acquired resistance.^7^^,^^8^ Clinical data also suggest that first-line EGFR-TKI–chemotherapy combination may provide better outcomes for patients with EGFRm NSCLC versus EGFR-TKIs alone. In two randomized phase III studies, progression-free survival and overall survival were statistically and clinically significantly longer with gefitinib–carboplatin–pemetrexed versus gefitinib monotherapy.^9^^,^^10^ Increased Common Terminology Criteria for Adverse Events (CTCAE) grade ≥3 toxicity, mostly hematologic toxicities or chemotherapy-induced nephrotoxicity, was reported in the combination group; however, these adverse events (AEs) were deemed manageable.^9^^,^^10^

The phase III FLAURA2 study (NCT04035486) will evaluate the efficacy and safety of first-line osimertinib in combination with platinum–pemetrexed chemotherapy versus osimertinib monotherapy in EGFRm advanced/metastatic NSCLC. Here, we report the results from the safety run-in of FLAURA2, assessing the safety and tolerability of osimertinib–chemotherapy prior to the randomized evaluation phase.

## Materials and methods

### Study design and treatments

Patients received oral osimertinib 80 mg once daily (QD) in combination with either intravenous (IV) cisplatin 75 mg/m^2^ or IV carboplatin target area under the curve 5, plus pemetrexed 500 mg/m^2^, both administered every 3 weeks (Q3W) for four cycles (21 days per cycle; Supplementary Figure S1, available at https://doi.org/10.1016/j.esmoop.2021.100271). Patients were allowed to switch to the alternative platinum chemotherapy at the investigator’s discretion, provided the patient discontinued the initial chemotherapy due to toxicity. This was followed by osimertinib 80 mg QD with pemetrexed 500 mg/m^2^ maintenance Q3W until RECIST 1.1-defined progression or discontinuation (patient/investigator decision, AE, protocol noncompliance).

### Objectives and assessments

The primary objective was to evaluate the safety and tolerability of osimertinib–chemotherapy. Secondary objectives were pharmacokinetic assessment of osimertinib–chemotherapy. Safety was evaluated using AEs graded by CTCAE version 5.0, and laboratory evaluations of clinical chemistry, hematology, urinalysis, vital signs, all at every visit; physical examinations were completed on day 1 of each cycle.

### Patients

We included patients aged ≥18 years (Japan: ≥20 years) with locally advanced/metastatic nonsquamous NSCLC and tumors harboring a locally or centrally confirmed EGFR-TKI sensitizing mutation (ex19del/L858R), either alone or in combination with other EGFR mutations, including T790M; World Health Organization performance status 0/1; and no prior therapy for advanced disease.

Key exclusion criteria included symptomatic and unstable CNS metastases except for patients who had completed definitive therapy, were not on steroids, and had a stable neurological status for ≥2 weeks after completion of the definitive therapy and steroids; history of clinically active interstitial lung disease; and evidence of severe/uncontrolled systemic disease.

### Standard protocol approvals, registration, and patient consents

The trial was conducted in accordance with the provisions of the Declaration of Helsinki, Good Clinical Practice guidelines (as defined by the International Conference on Harmonization), applicable regulatory requirements, and the Policy on Bioethics and Human Biologic Samples of the trial sponsor, AstraZeneca. All patients provided written informed consent.

### Statistical analyses

Thirty patients (15 per treatment group) were planned to be evaluated in a nonrandomized fashion. Safety analysis was carried out in the safety analysis set (all patients who received ≥1 dose of any study treatment). No formal statistical testing was carried out.

The primary safety run-in analysis was to occur when ≥12 patients in each group had received three or more cycles of study treatment (representing ≥80% of patients completing ≥75% of planned treatment) or had discontinued study treatment due to unacceptable toxicity; data were reviewed by a safety review committee. Patients who participated here were not included in the randomized phase, but could continue their allocated treatment. Data cut-off (DCO) date: 19 February 2020.

## Results

### Patient disposition and demographics

Of 43 patients enrolled, 13 were screen failures and 30 received treatment (15 per group). At DCO, 13 patients (87%) receiving osimertinib–carboplatin–pemetrexed and 10 patients (67%) receiving osimertinib–cisplatin–pemetrexed had completed the protocol-defined four chemotherapy cycles. Most patients were Asian (73%) and female (63%), with a median age (range) of 61 (45-84) years (Table 1). Baseline demographics and disease characteristics were generally similar between the groups, other than a higher proportion of Asian patients and never-smokers receiving osimertinib–carboplatin–pemetrexed versus osimertinib–cisplatin–pemetrexed (Table 1).

**Table 1:** Baseline demographics and clinical characteristics

Characteristic, n (%)	Osimertinib–carboplatin–pemetrexed (*n* = 15)	Osimertinib–cisplatin–pemetrexed (*n* = 15)	Total (*n* = 30)
Sex			
Male	6 (40)	5 (33)	11 (37)
Female	9 (60)	10 (67)	19 (63)
Median age (range), years	61 (45-84)	60 (48-72)	61 (45-84)
Race			
Asian	13 (87)	9 (60)	22 (73)
White	2 (13)	6 (40)	8 (27)
Smoking status			
Never	12 (80)	7 (47)	19 (63)
Current	0 (0)	0 (0)	0 (0)
Former	3 (20)	8 (53)	11 (37)
World Health Organization performance status			
0	6 (40)	7 (47)	13 (43)
1	9 (60)	8 (53)	17 (57)
EGFR mutation at entry^a^			
Ex19del	10 (67)	10 (67)	20 (67)
L858R	5 (33)	5 (33)	10 (33)
Overall disease			
Metastatic	15 (100)	15 (100)	30 (100)
Metastases			
Central nervous system	3 (20)	2 (13)	5 (17)
Liver	2 (13)	5 (33)	7 (23)
Adenocarcinoma histology	15 (100)	15 (100)	30 (100)

Note: Data are *n* (%) unless otherwise stated.

EGFR, epidermal growth factor receptor.

a EGFR test used for enrollment: 27 patients (90%) enrolled using a local EGFR test; three patients (10%) enrolled using central cobas EGFR test; one patient (3%) did not have ex19del or L858R confirmed by a central cobas EGFR test.

### Safety

**Figure 1:**
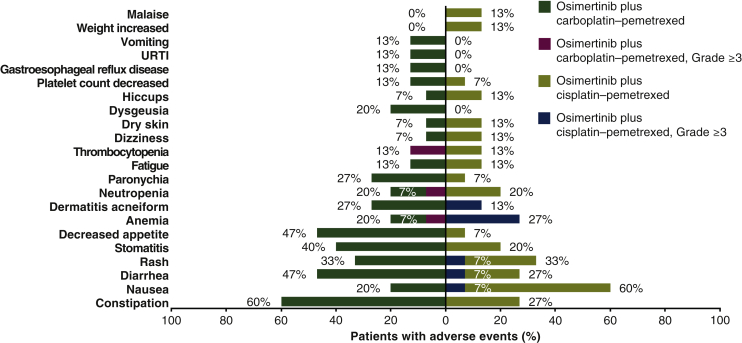
Most common adverse events (AEs) occurring in >10% of study population with any treatment and AEs with Common Terminology Criteria for Adverse Events (CTCAE) grade ≥3. AE order follows the frequency in the overall study population. URTI, upper respiratory tract infection.

At DCO, median (Range) duration of osimertinib exposure was 3.81 (1.9-7.1) months, and duration of pemetrexed exposure was 4.14 (0.7-7.6) months. Median duration of carboplatin and cisplatin exposure was similar [2.76 (0.7-3.2) months and 2.79 (0.7-3.3) months, respectively]. AEs were reported in 27 patients (90%; Table 2). The most common AE (any grade) with osimertinib–carboplatin–pemetrexed was constipation (60%), and with osimertinib–cisplatin–pemetrexed it was nausea (60%; Figure 1).

**Table 2:** Safety summary

Patients, *n* (%)	Osimertinib–carboplatin–pemetrexed (*n* = 15)	Osimertinib–cisplatin–pemetrexed (*n* = 15)	Total (*n* = 30)
Any AE	15 (100)	12 (80)	27 (90)
AE causally related to any treatment^a^	15 (100)	12 (80)	27 (90)
Osimertinib	14 (93)	8 (53)	22 (73)
Carboplatin–cisplatin	13 (87)	12 (80)	25 (83)
Pemetrexed	12 (80)	10 (67)	22 (73)
AE of CTCAE grade ≥3	3 (20)	8 (53)	11 (37)
AE of CTCAE grade ≥3, causally related to any treatment^a^^,^^b^	3 (20)	7 (47)	10 (33)
Osimertinib	0 (0)	2 (13)	2 (7)
Carboplatin–cisplatin	3 (20)	6 (40)	9 (30)
Pemetrexed	3 (20)	6 (40)	9 (30)
SAE	3 (20)	3 (20)	6 (20)
SAE, causally related to any treatment^a^	1 (7)	2 (13)	3 (10)
Osimertinib	0 (0)	0 (0)	0 (0)
Carboplatin–cisplatin	1 (7)	2 (13)	3 (10)
Pemetrexed	1 (7)	1 (7)	2 (7)
AE leading to discontinuation of any study drug	4 (27)	3 (20)	7 (23)
Osimertinib	1^c^ (7)	0 (0)	1 (3)
Carboplatin–cisplatin	2 (13)	2^d^ (13)	4 (13)
Pemetrexed	3 (20)	3 (20)	6 (20)
AE leading to dose reduction of any study drug	1 (7)	1 (7)	2 (7)
Osimertinib	0 (0)	1 (7)	1 (3)
Carboplatin–cisplatin	0 (0)	0 (0)	0 (0)
Pemetrexed	1 (7)	0 (0)	1 (3)
AE leading to dose interruption of any study drug	3 (20)	2 (13)	5 (17)
Osimertinib	2 (13)	2 (13)	4 (13)
Carboplatin–cisplatin	2 (13)	0 (0)	2 (7)
Pemetrexed	3 (20)	0 (0)	3 (10)
AE with outcome of death	1 (7)	0 (0)	1 (3)
AE with outcome of death, causally related to treatment^a^	0 (0)	0 (0)	0 (0)

Note: All AEs occurring after the first dose and within 28 days of discontinuation of the last dose of study treatment but prior to start of a new anticancer treatment were included. One patient in the osimertinib–cisplatin–pemetrexed group discontinued all study treatments due to patient decision, not related to treatment.

CTCAE, Common Terminology Criteria for Adverse Events; AE, adverse event; SAE, severe adverse event.

a As assessed by the investigator.

b Reported AEs CTCAE grade ≥3 by preferred term (a patient could report more than one AE): osimertinib–carboplatin–pemetrexed group, anemia (*n* = 1), neutropenia (*n* = 1), thrombocytopenia (*n* = 2), leukopenia (*n* = 1), neutrophil count decreased (*n* = 1); osimertinib–cisplatin–pemetrexed group, anemia (*n* = 3), neutropenia (*n* = 2), diarrhea (*n* = 1), nausea (*n* = 1), inappropriate antidiuretic hormone secretion (*n* = 1), pyrexia (*n* = 1), pulmonary embolism (*n* = 1), and rash (*n* = 1).

c In total, two patients discontinued all study treatments, including osimertinib, but one patient died due to a fatal AE not related to study treatment and is therefore not included in this table.

d One patient switched from cisplatin to carboplatin after one cycle and completed all four cycles of chemotherapy.

The number of patients reporting serious AEs (SAEs) was the same for both treatment groups (three patients each). Three of the six patients reporting SAEs each had an event considered treatment related by the investigator: nausea related to carboplatin–pemetrexed (recovered, no change to treatment); pulmonary embolism related to cisplatin–pemetrexed (not recovered, no change to treatment); and cisplatin-induced hyponatremia (recovered with sequelae; switched to carboplatin).

With osimertinib–carboplatin–pemetrexed, none of the patients required an osimertinib dose reduction and two patients (13%) had an osimertinib dose interruption that did not lead to discontinuation (AEs: injury from falling, decreased neutrophil count). With osimertinib–cisplatin–pemetrexed, one patient (7%) required an osimertinib dose reduction (AEs of pyrexia, hyponatremia, rash) and two patients (13%) required osimertinib dose interruption (AEs: diarrhea, rash).

AEs leading to discontinuation of any study drug occurred in seven patients (23%; Table 2; Supplementary Figure S2, available at https://doi.org/10.1016/j.esmoop.2021.100271). One patient (osimertinib–carboplatin–pemetrexed; 3%) discontinued all study treatments due to pneumonitis (protocol-defined discontinuation criterion; grade 2); following discontinuation the patient recovered. One patient receiving osimertinib–carboplatin–pemetrexed who discontinued carboplatin and pemetrexed due to thrombocytopenia (grade 3) had a fatal AE of hemoptysis that was considered unrelated to study treatment by the investigator; osimertinib treatment was ongoing at time of death, and death was attributed to NSCLC.

In the total study population, 11 patients (37%) reported AEs of CTCAE grade ≥3; in ten patients (33%) these were considered causally related to any treatment (Table 2). No clinically relevant changes in laboratory evaluations, vital signs, or physical findings were observed. Eight of the 11 patients reported 12 grade ≥3 hematologic AEs [a patient could have reported more than one AE; osimertinib–carboplatin–pemetrexed, *n* = 3, osimertinib–cisplatin–pemetrexed, *n* = 5; outcomes: fully recovered, *n* = 4; ongoing events, *n* = 3; died (carboplatin group, described previously), *n* = 1]. In seven patients (23%) these were considered related to treatment and stabilized under continued study treatment.

## Discussion

The FLAURA2 safety run-in evaluated 30 patients dosed with osimertinib in combination with platinum-based chemotherapy. Osimertinib combined with chemotherapy was generally well tolerated and most AEs were mild to moderate in severity. No specific pattern of AEs was associated with dose modifications or discontinuations. In general, patients continued to receive osimertinib; only one patient discontinued all study treatments, including osimertinib, due to an AE of pneumonitis. One patient died and was consequently considered discontinued from all treatments.

The safety findings observed, including the most commonly reported AEs, were consistent with the known toxicities of osimertinib monotherapy and platinum–pemetrexed chemotherapy, and there was no evidence of additive or emerging toxicities with the combination therapy. They were also in line with previous phase II and III studies investigating EGFR-TKIs plus chemotherapy.^9^, ^10^, ^11^, ^12^ Hematologic toxicities were reported, but were within the scope of toxicities expected for chemotherapy agents, and were manageable by standard clinical practice, with evidence of reversibility. No clear differences in safety or tolerability were observed between the two chemotherapy regimens. In addition, exposure to osimertinib and metabolite AZ5104 was similar when coadministered with carboplatin or cisplatin plus pemetrexed. Osimertinib exposure was within the range observed in monotherapy studies; no obvious or clinically significant interaction was noted.^13^ These initial data support the continued investigation of osimertinib and chemotherapy combinations in the phase III FLAURA2 randomized trial.

The current FLAURA2 safety run-in data, potentially together with initial data from the presently recruiting FLAURA2 randomized trial, may also help inform safety considerations regarding combination treatment in the phase III randomized COMPEL trial (NCT04765059). COMPEL is due to investigate the use of platinum–pemetrexed chemotherapy with continued osimertinib in patients who experience non-CNS progression on first-line osimertinib.

The FLAURA2 trial is not designed to investigate the impact of specific concomitant mutations (e.g. *TP53, RB1*) which can be present in patients with *EGFR* mutations that are treated with osimertinib. However, there is increasing interest regarding the effect of such concomitant mutations on the efficacy of EGFR-TKIs.^14^^,^^15^ These concomitant mutations may explain, in part, the clinical heterogeneity that can be observed in response to first-line EGFR-TKIs, including osimertinib.^14^^,^^16^^,^^17^ The phase III TOP study (NCT04695925) will compare efficacy and safety of osimertinib with osimertinib–pemetrexed–carboplatin combination in treatment-naïve patients with advanced NSCLC with concurrent *EGFR* and *TP53* mutations. It may be interesting to consider the results of the TOP study alongside those of FLAURA2 when available.

Limitations of this FLAURA2 safety run-in study included the small population size, short duration of exposure by DCO, and no osimertinib monotherapy comparator arm (an osimertinib monotherapy comparator arm will be included in the phase III randomized period).

### Conclusions

In conclusion, the FLAURA2 safety run-in indicated that the osimertinib–chemotherapy combination had a manageable safety and tolerability profile in patients with EGFRm advanced/metastatic NSCLC, supporting further assessment of this first-line combination in the phase III randomized period. Approximately 556 patients will be randomized 1:1 (osimertinib–chemotherapy:osimertinib monotherapy) to enable formal efficacy testing and further safety evaluation. Preliminary results are expected in 2023.
