# Supplementary material for: Osimertinib plus platinum–pemetrexed in newly diagnosed epidermal growth factor receptor mutation-positive advanced/metastatic non-small-cell lung cancer: safety run-in results from the FLAURA2 study
Source: ESMO Open. 2021 Sep 17;6(5):100271. doi: 10.1016/j.esmoop.2021.100271 (PMC8453202; doi:10.1016/j.esmoop.2021.100271)
Supplement: Supplemental Material [file mmc1.docx]

**Supplemental material**

**Figure S1. FLAURA2 safety run-in study design.**

**
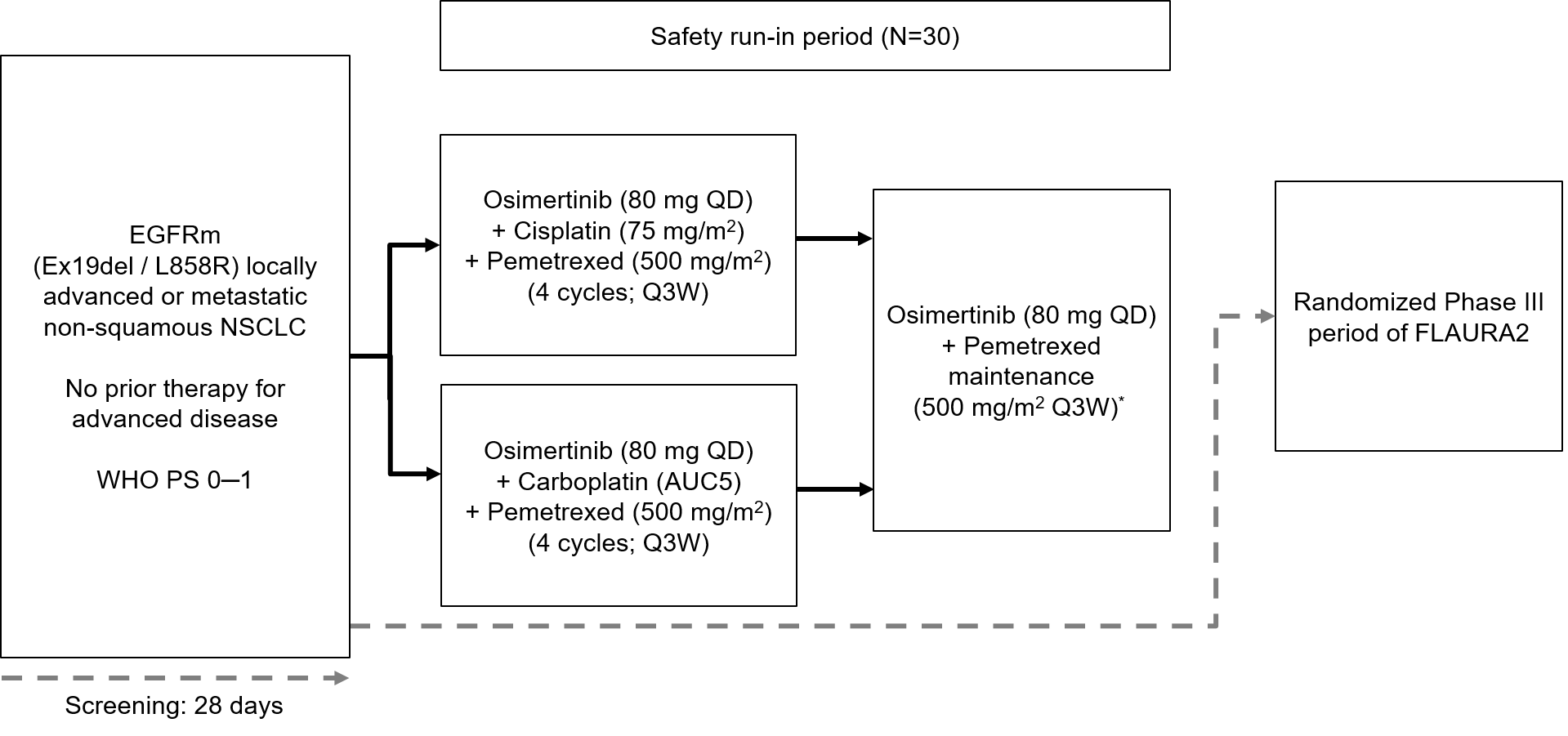
**

*Maintenance to continue until RECIST 1.1-defined progression or another discontinuation criterion is met.
AUC5, area under the curve 5; EGFRm, epidermal growth factor receptor mutation positive; Ex19del, exon 19 deletion; NSCLC, non-small cell lung cancer; PS, performance status; Q3W, every three weeks; QD, daily; WHO, World Health Organization.

**Figure S2. Discontinuation of any study treatment due to AEs.**

**
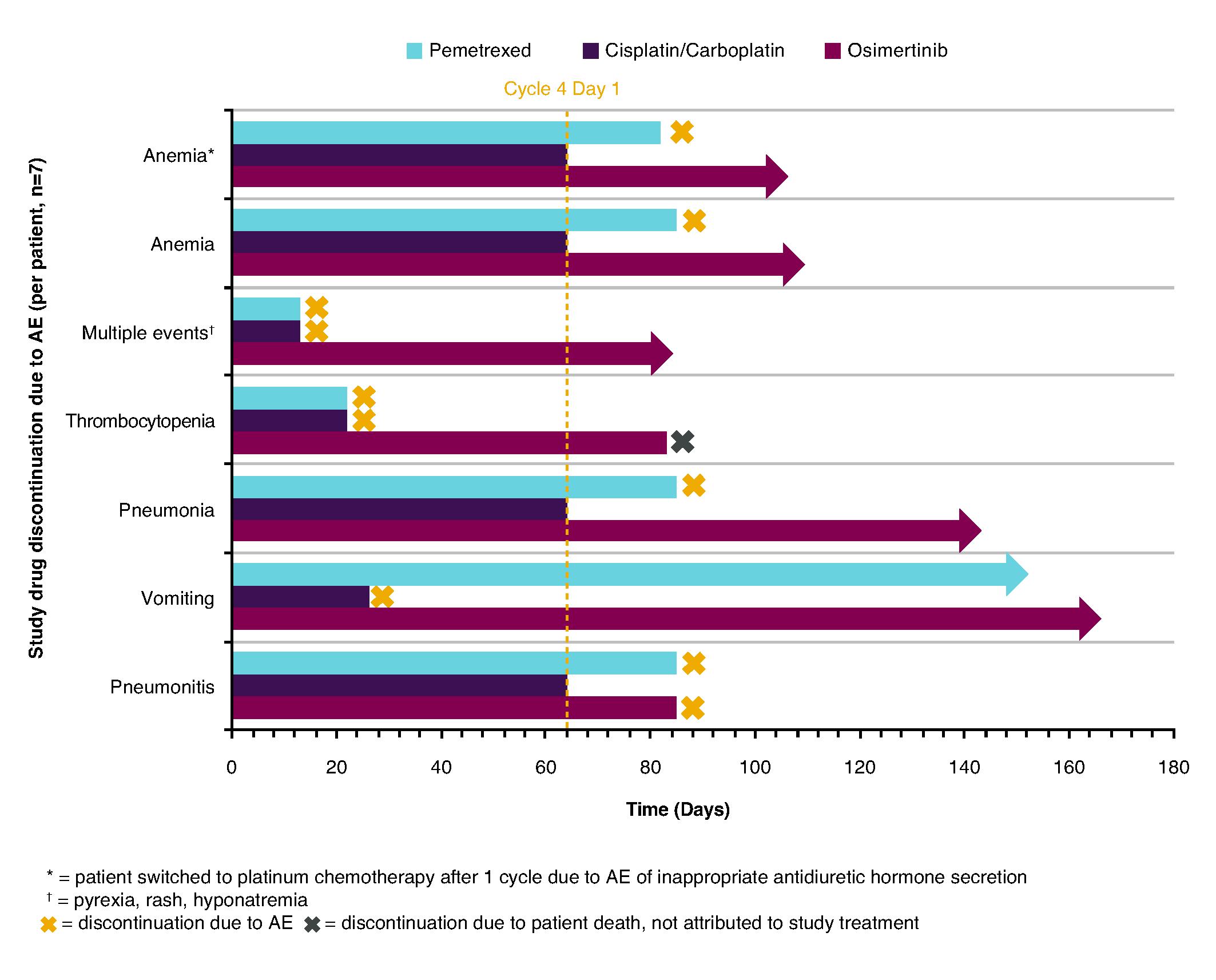
**

Figure presented at ESMO 2020 and ESMO Asia 2020. AE, adverse event.
